# Clinical importance of VEGFC and PD‐L1 co‐expression in lung adenocarcinoma patients

**DOI:** 10.1111/1759-7714.13354

**Published:** 2020-03-10

**Authors:** Tingting Qin, Junling Xia, Shaochuan Liu, Jing Wang, Hailin Liu, Yan Zhang, Yanan Jia, Kai Li

**Affiliations:** ^1^ Tianjin Medical University Cancer Institute and Hospital National Clinical Research Center for Cancer Tianjin China; ^2^ Key Laboratory of Cancer Prevention and Therapy Tianjin China; ^3^ Tianjin's Clinical Research Center for Cancer Tianjin China; ^4^ Department of Thoracic Oncology, Tianjin Lung Cancer Center, Tianjin Cancer Institute & Hospital Tianjin Medical University Tianjin China; ^5^ Department of Biomedical Engineering Tianjin Medical University Tianjin China

**Keywords:** Lung adenocarcinoma, overall survival, PD‐L1, progression‐free survival, VEGFC

## Abstract

**Background:**

Vascular endothelial growth factor C (VEGFC), an activator of lymphangiogenesis, is newly identified as an immunomodulator which can regulate the immune system so that tumor cells more easily escape immune surveillance. Evidence has shown programmed cell death‐ligand 1 (PD‐L1) can also suppress the immune response. Nevertheless, the clinical significance of co‐expression of VEGFC and PD‐L1 for predicting outcomes in patients with lung adenocarcinoma has not yet been determined.

**Methods:**

A total of 114 patients with lung adenocarcinoma who underwent surgeries at Tianjin Medical University Cancer Institute and Hospital between December 2011 and September 2016 were retrospectively reviewed. Tissue specimens were collected for immunohistochemistry of VEGFC and PD‐L1 which were analyzed with an H‐score system.

**Results:**

In this study, 57 (50.0%) and 47 (41.2%) patients were classified as VEGFC high expression and PD‐L1 high expression. Co‐expression was observed in 33 (28.9%) patients. In addition, a positive correlation was found between VEGFC and PD‐L1 (*P* = 0.0398, r = 0.1937). In a univariate analysis, both progression‐free survival (PFS) and overall survival (OS) were significantly worse in the VEGFC high expression group and the PD‐L1 high expression group, respectively. Furthermore, VEGFC/PD‐L1 co‐expression showed a worse OS (*P* = 0.03) and PFS survival (*P* = 0.01) than the other groups.

**Conclusions:**

Taken together, these results indicate that VEGFC/PD‐L1 co‐expression can forecast both poor OS and PFS in patients with resected lung adenocarcinoma. Co‐expression of VEGFC and PD‐L1 may serve as a significant prognostic factor for patients with lung adenocarcinoma.

**Key points:**

VEGFC/PD‐L1 co‐expression forecasts poor survival in patients with resected lung adenocarcinoma. VEGFC/PD‐L1 co‐expression may be used as a prognostic indicator and provide the theoretical possibility to screen the optimal population with a combination of anti‐VEGFC and anti‐PD‐L1 therapy in the clinical treatment.

## Introduction

Lung cancer is recognized as the leading cause of cancer‐related death worldwide.^1^ Lung adenocarcinoma is the most common subtype^2^ in non‐small cell lung cancer (NSCLC), which accounts for 80%–85% of all lung cancer patients. As is known to all, targeted therapies for mutant driver genes have improved clinical outcomes in certain patients.^3^ More recently, immunotherapy has emerged as an exciting alternative treatment for patients without an actionable driver mutation.^4^ However, the five‐year survival rate for lung adenocarcinoma patients still remains unsatisfactory, partly because cancer immunotherapy is not completely effective in eradicating tumor cells because they escape from host immune scrutiny. To improve the efficacy of immunotherapy, there is an urgent need to find ideal immune‐associated biomarkers to accurately assess the clinical decision, progression‐free survival (PFS) and overall survival (OS) of patients with lung adenocarcinoma.

Vascular endothelial growth factor C (VEGFC), the classical lymphangiogenic factor, which acts mainly in developmental‐ and disease‐associated lymphangiogenesis, has been newly identified as an immunomodulator which can regulate the immune system so that tumor cells more easily escape immune surveillance.^5^ Tacconi *et al*. reported VEGFC could enhance tumor growth via fostering cancer immune escape.^6^ Lund *et al*. demonstrated VEGFC could promote immune tolerance via suppression of CD8^+^ T cells.^7^ In addition, high expression of VEGFC has been reported to be significantly associated with poor prognosis in a variety of malignancies.^8^, ^9^, ^10^, ^11^, ^12^, ^13^

Programmed cell death‐ligand 1 (PD‐L1), which is widely expressed in immune cells, lymphatic endothelial cells, blood endothelial cells, tumor cells and so on, can suppress immune‐response.^14^, ^15^ High expression of PD‐L1 is associated with an unfavorable survival in patients with lung cancer.^16^

In the present study, we summarize the data of clinical characteristics of 114 cases of lung adenocarcinoma patients, explore the relationship between VEGFC and PD‐L1 expression in patients with resected lung adenocarcinoma, and search for their predictive value in future immunotherapy for patients with lung adenocarcinoma.

## Methods

### Patients

A total of 114 patients diagnosed with lung adenocarcinoma (52 with wild‐type, 48 with *EGFR* mutations, 10 with *KRAS* mutations and four patients with *ALK* mutations) were included in the study. All tumor samples were surgically resected in Tianjin Medical University Cancer Institute and Hospital from December 2011 to September 2016. The Ethics Committee of the Tianjin Medical University Cancer Institute and Hospital (Tianjin, China) approved the use of human tissues for this study (EK2018039). The study conforms to recognized standards of the Declaration of Helsinki and its outcomes will not affect the future management of the patients. Each patient signed an informed consent. Inclusion criteria were as follows: (i) Patients with an exact date of follow‐up; (ii) no neoadjuvant treatment had been carried out before surgery; (iii) patients were stage I to stage III (AJCC/UICC TNM Classification and stage groupings). Clinical characteristics of the patients included age, gender, smoking status, gene mutation status, histological subtypes, clinical stage, postoperative treatments, PFS and OS. The clinical follow‐up information was obtained from patients’ medical records.

### Immunohistochemical staining

The 4 μm thick, formalin‐fixed, paraffin‐embedded tumors of clinical specimens of lung adenocarcinoma were deparaffinized in xylene and rehydrated in a graded series of alcohols, then rinsed three times with PBS. Antigen retrieval was performed in the pressure cooker at 130°C for three minutes, citrate buffer (PH 6.0) was used for VEGFC staining, and EDTA solution (PH 11.0) was used for PD‐L1 staining. The slides were then incubated in 3% H_2_O_2_ for 15 minutes. For immunohistochemical staining the slides were incubated with primary antibodies against VEGFC (ab135506, Abcam, USA), 1:100, or against PD‐L1/CD274 (66 248, Proteintech, USA), 1:1200, at 4°C, overnight. Incubation of secondary antibody and coloration were then carried out by EIVISON plus (kit‐9903, MXB, China) and DAB kit (ZL1‐9019, ZSGB‐BIO, China), respectively. Counterstain was performed with hematoxylin for two minutes. Three clinical pathologists assessed the intensity of the immunostaining on each section independently in a blinded manner. At least 10 fields per specimen were surveyed.

### Immunohistochemical staining analysis

VEGFC expression and PD‐L1 expression in this study were scored with an H‐score system (ranging from 0 to 300). Its specific calculation method was the sum of the intensity of staining (0 was negative; 1 was weak positive; 2 was moderate positive; 3 was strong positive) and the percentage of positive tumor cells (0%–100%, with any intensity of positive tumor cell staining).^17^ Two clinical pathologists graded the scores of each slide independently in a blinded manner. When considering the VEGFC expression,^18^ the cutoff value was set at 100, ie. H‐score > 100 was identified as a VEGFC high expression case. According to previous studies,^19^, ^20^ PD‐L1 cutoff value was set at 100, ie. H‐score >100 was considered to be a PD‐L1 high expression case.

### Statistical analysis

**Figure 1:**
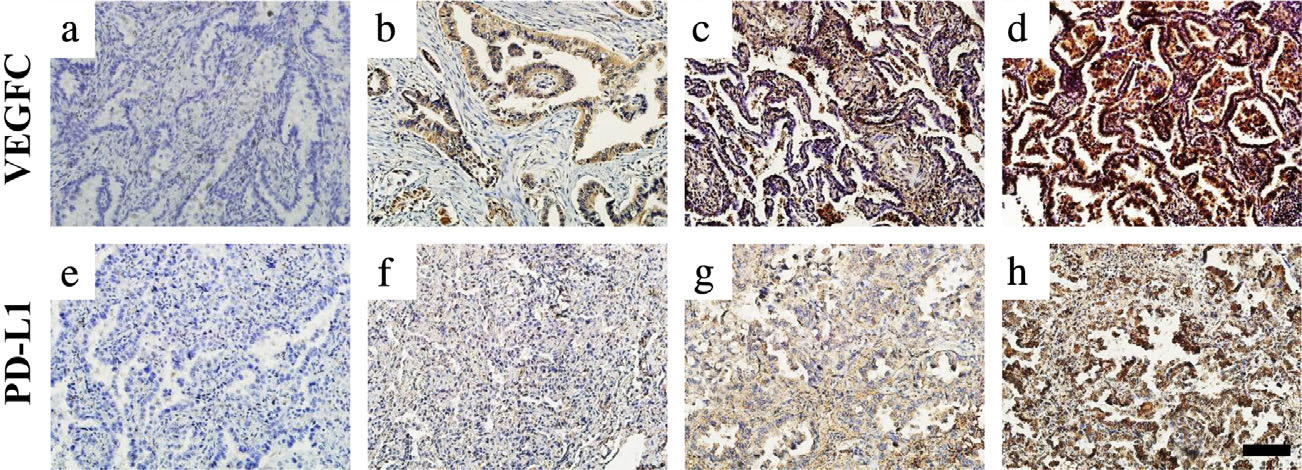
Representative images of immunohistochemical staining for VEGFC and PDL1 expression in lung adenocarcinoma. (**a**) VEGFC negative expression; (**b**) VEGFC weak positive expression; (**c**) VEGFC moderate positive expression; (**d**) VEGFC strong positive expression; (**e**) PD‐L1 negative expression; (**f**) PD‐L1 weak positive expression; (**g**) PD‐L1 moderate positive expression; (**h**) PD‐L1 strong positive expression; scale bar: 100 μm.

SPSS v.21 (IBM Corp, Armonk, NY, USA) and GraphPad Prism 6 (USA, GraphPad Software) were used for statistical analyses, and survival curve, respectively. Fisher's exact test was performed to compare the correlations between VEGFC/PD‐L1 expression (Fig 1) and clinical features. Pearson's correlation coefficient test was used to determine the relationship between the expression of VEGFC and expression of PD‐L1. Kaplan‐Meier survival curve and log‐rank test were used to estimate and compare the survival of lung adenocarcinoma patients in different groups. Multivariate and univariate analysis were carried out. A two‐tailed *P*‐value <0.05 was considered statistically significant.

## Results

### Clinicopathological characteristics in patients with lung adenocarcinoma

A total of 114 patients with lung adenocarcinoma were included in our study cohort. The clinicopathological characteristics are shown in Table 1. There were 52 (45.6%) patients who were male, 48 (42.1%) patients were over 60‐years‐old, and 66 (57.9%) were smokers. At diagnosis, there were 83 (72.8%) stage I/II patients, 76 (66.7%) with acinar adenocarcinoma, and a total of 48 (42.1%) cases and 14 (12.3%) patients with *EGFR* mutations and other mutations, including *KRAS* and *ALK*, respectively. There were 57 (50.0%) and 47 (41.2%) patients with high VEGFC expression and high PD‐L1 expression, respectively. There were 42 (36.8%) patients in the VEGFC^−‐^& PD‐L1^−‐^ group, 24 (21.1%) patients in the VEGFC^+^& PD‐L1^−‐^ group, 15 (13.2%) patients in the VEGFC^−‐^&PD‐L1^+^ group and 33 (28.9%) patients in the VEGFC^+^& PD‐L1^+^ group (Table S1). However, no significant correlation was observed for VEGFC or PD‐L1 expression and other clinicopathological characteristics such as gender, age, smoking status, stage, histological subtypes, T factor, N factor, or gene status (all *P* > 0.05) (Table 2).

**Table 1:** Clinical characteristics of 114 patients with lung adenocarcinoma

Characteristics	ALL, n (%)
Gender	
Male	52 (45.6)
Female	62 (54.4)
Age	
<60	66 (57.9)
≥60	48 (42.1)
Smoking history	
Yes	66 (57.9)
No	48 (42.1)
Stage	
I	69 (60.5)
II	14 (12.3)
III	31 (27.2)
Histological subtypes	
Lepidic predominant	20 (17.5)
Acinar predominant	76 (66.7)
Papillary/micropapillary predominant	9 (7.9)
Solid predominant	3 (2.6)
Other	6 (5.3)
T factor	
T1	69 (60.5)
T2	14 (12.3)
T3	31 (27.2)
N factor	
N0	78 (68.4)
N1	6 (5.3)
N2	30 (26.3)
Gene status	
WT†	52 (45.6)
EGFR‡	48 (42.1)
Other mutations	14 (12.3)
VEGFC§	
H‐score≤100	57 (50.0)
H‐score>100	57 (50.0)
PD‐L1¶	
H‐score ≤100	67 (58.8)
H‐score >100	47 (41.2)
Postoperative therapy	
Treatment	75 (65.8)
Nontreatment	39 (34.2)

† WT, wild‐type.

‡ EGFR, epidermal growth factor receptor.

§ VEGFC, Vascular endothelial growth factor C.

¶ PD‐L1, programmed cell death‐ligand 1.

**Table 2:** Relationship between PD‐L1/VEGFC expression and the clinical characteristics in 114 patients with lung adenocarcinoma (SEM)

Characteristics	PD‐L1 negative, n (%)	PD‐L1 positive, n (%)	*P*‐value	VEGFC negative, n (%)	VEGFC positive, n (%)	*P*‐value
Gender			0.583			0.452
Male	32 (47.8)	20 (42.6)		28 (49.1)	24 (42.1)	
Female	35 (52.2)	27 (57.4)		29 (50.9)	33 (57.9)	
Age			0.216			0.448
<60	42 (62.7)	24 (51.1)		31 (54.4)	35 (61.4)	
≥60	25 (37.3)	23 (48.9)		26 (45.6)	22 (38.6)	
Smoking history			0.282			0.448
Yes	36 (53.7)	30 (63.8)		31 (54.4)	35 (61.4)	
No	31 (46.3)	17 (36.2)		26 (45.6)	22 (38.6)	
Stage			0.401			0.626
I	43 (64.2)	26 (55.3)		32 (56.1)	37 (64.9)	
II	6 (9.0)	8 (17.0)		8 (14.1)	6 (10.5)	
III	18 (26.8)	13 (27.7)		17 (29.8)	14 24.6)	
Histological types			0.284			0.116
Lepidic predominate	24 (35.8)	17 (36.2)		23 (40.3)	18 (31.6)	
Acinar predominate	23 (34.3)	11 (23.4)		12 (21.1)	22 (38.6)	
Papillary/micropapillary predominate	8 (11.9)	13 (27.7)		9 (15.8)	12 (21.0)	
Solid predominate	5 (7.5)	1 (2.1)		4 (7.0)	2 (3.5)	
Other	7 (10.5)	5 (10.6)		9 (15.8)	3 (5.3)	
T			0.200			0.624
T1	39 (58.2)	28 (59.6)		33 (57.9)	34 (59.7)	
T2	24 (35.8)	12 (25.5)		17 (29.8)	19 (33.3)	
T3	4 (6.0)	7 (14.9)		7 (12.3)	4 (7.0)	
**N**			0.669			0.670
N0	48 (71.6)	30 (63.8)		39 (68.4)	39 (68.4)	
N1	3 (4.5)	3 (6.4)		4 (7.0)	2 (3.5)	
N2	16 (23.9)	14 (29.8)		14 (24.6)	16 (28.1)	
Gene status			0.280			0.070
WT	32 (47.8)	20 (42.6)		26 (45.6)	26 (45.6)	
EGFR	23 (34.3)	25 (53.2)		25 (43.9)	23 (40.4)	
Other mutations	12 (17.9)	2 (4.2)		6 (10.5)	8 (14.0)	
Postoperative therapy			0.712			0.843
Treatment	45 (67.2)	30 (63.8)		37 (64.9)	38 (66.7)	
Nontreatment	22 (32.8)	17 (36.2)		20 (35.1)	19 (33.3)	
VEGFC			**0.001** ***			
H‐score ≤ 100	25 (37.3)	32 (68.1)				
H‐score > 100	42 (62.7)	15 (31.9)				

*** P=0.001.

### Analysis of patient survival

**Figure 2:**
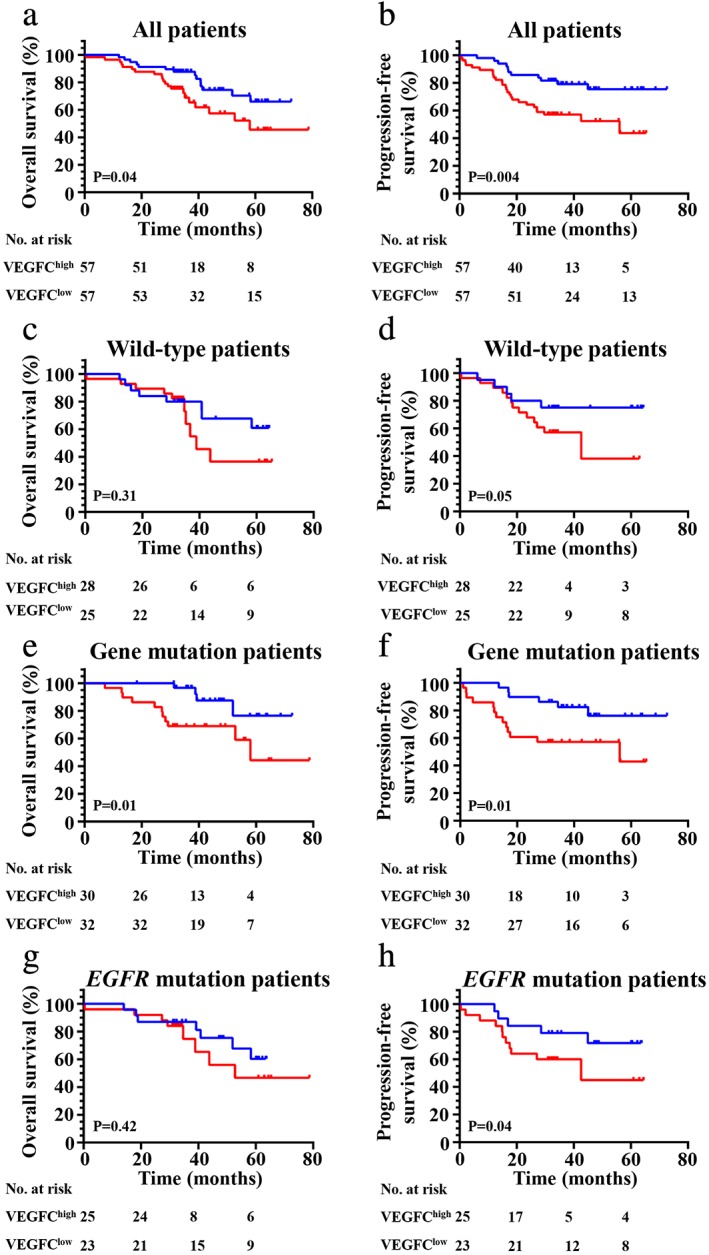
Kaplan‐Meier curves show (**a**, **c, e** and **g**) OS and (**b**, **d**, **f** and **h**) PFS of different groups (all patients, wild‐type patients, gene mutation patients or *EGFR* mutation patients, respectively) with high and low expression of VEGFC. (**a**) (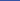) VEGFC^low^ (*N* = 57, 14 events), and (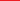) VEGFC^high^ (*N* = 57, 21 events); (**b**) (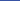) VEGFC^low^ (*N* = 57, 11 events), and (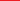) VEGFC^high^ (*N* = 57, 26 events); (**c**) (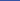) VEGFC^low^ (*N* = 25, eight events), and (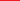) VEGFC^high^ (*N* = 28, 10 events); (**d**) (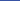) VEGFC^low^ (*N* = 25, five events), and (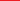) VEGFC^high^ (*N* = 18, 13 events); (**e**) (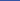) VEGFC^low^ (*N* = 32, four events), and (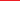) VEGFC^high^ (*N* = 30, 11 events); (**f**) (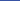) VEGFC^low^ (*N* = 32, six events), and (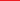) VEGFC^high^ (*N* = 30, 13 events); (**g**) (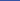) VEGFC^low^ (*N* = 23, seven events), and (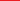) VEGFC^high^ (*N* = 25, eight events); (**h**) (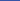) VEGFC^low^ (*N* = 23, five events), and (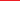) VEGFC^high^ (*N* = 25, 11 events).

**Figure 3:**
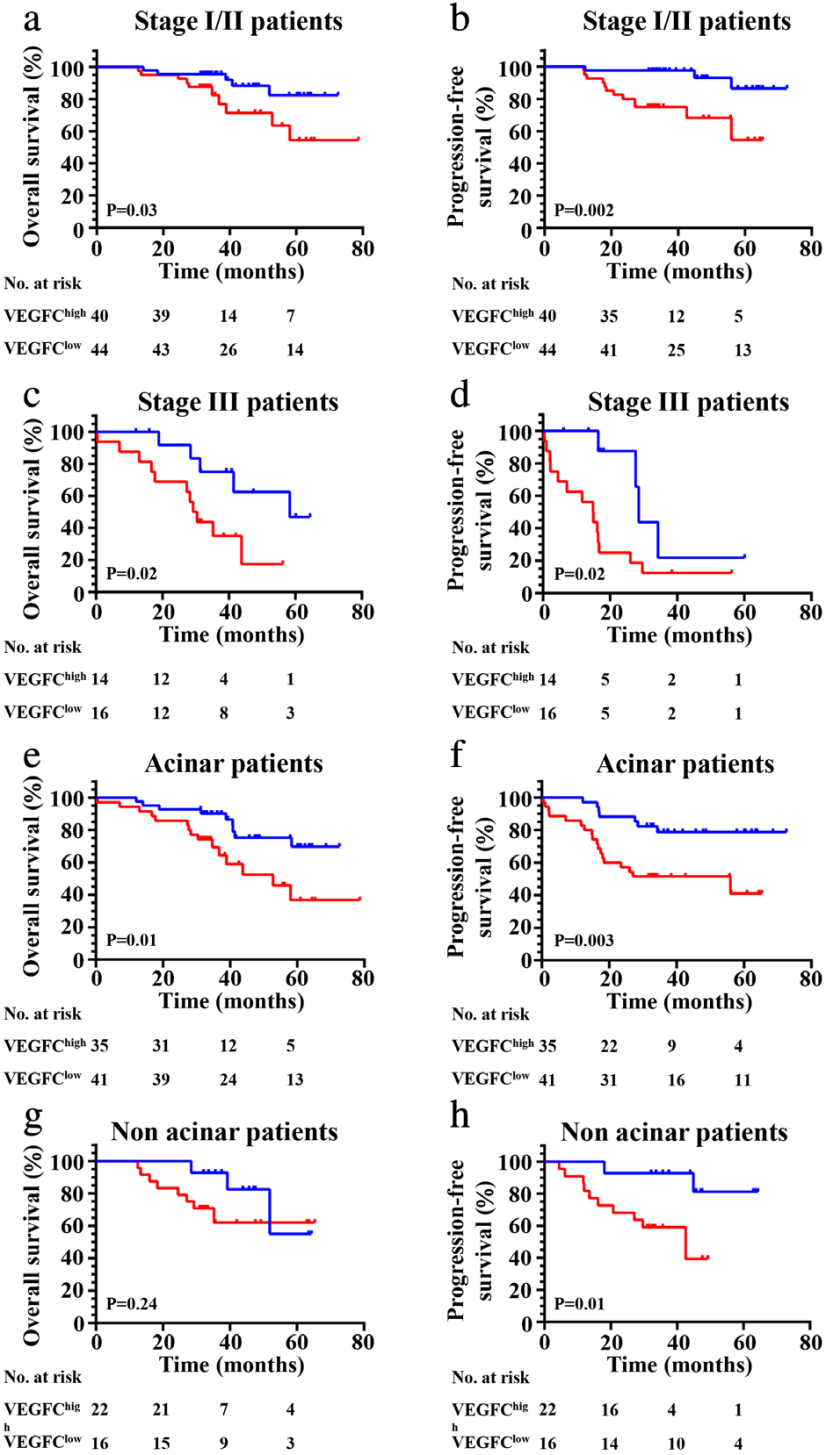
Kaplan‐Meier curves show (**a**, **c**, **e** and **g**) OS and (**b**, **d**, **f** and **h**) PFS of different subgroups (clinical stage I/II, III acinar or nonacinar adenocarcinoma, respectively) with high and low expression of VEGFC. (**a**) (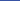) VEGFC^low^ (*N* = 44, five events), and (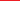) VEGFC^high^ (*N* = 40, 10 events); (**b**) (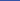) VEGFC^low^ (*N* = 44, three events), and (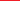) VEGFC^high^ (*N* = 40, 12 events); (**c**) (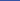) VEGFC^low^ (*N* = 16, five events), and (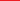) VEGFC^high^ (*N* = 14, 11 events); (**d**) (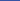) VEGFC^low^ (*N* = 16, four events), and (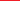) VEGFC^high^ (*N* = 14, 14 events); (**e**) (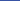) VEGFC^low^ (*N* = 41, nine events), and (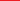) VEGFC^high^ (*N* = 35, 15 events); (**f**) (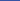) VEGFC^low^ (*N* = 41, seven events), and (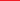) VEGFC^high^ (*N* = 35, 18 events); (**g**) (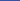) VEGFC^low^ (*N* = 16, three events), and (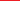) VEGFC^high^ (*N* = 22, eight events); (**h**) (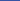) VEGFC^low^ (*N* = 16, two events), and (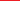) VEGFC^high^ (*N* = 22, 10 events).

In all patients, the VEGFC high expression group exhibited a significantly worse impact on the OS (*P* = 0.04) (Fig 2a) and PFS (*P* = 0.004) (Fig 2b). Further investigation was performed to analyze the correlation between VEGFC expression and survival in different subgroups. First, when considering patients in the wild‐type (WT) subgroup, no significant effect was seen on OS in the high VEGFC expression group and the low VEGFC expression group (Fig 2c); however, high VEGFC expression showed a poor PFS (*P* = 0.05) compared to low VEGFC expression (Fig 2d). Second, we found high VEGFC expression was significantly correlated with OS (*P* = 0.01) (Fig 2e) and PFS (*P* = 0.01) (Fig 2f) in all patients with any gene mutations. We then found that high VEGFC expression had no significant effect on OS (*P* = 0.42) (Fig 2g) but had a significant impact on PFS (*P* = 0.04) (Fig 2h) in patients with *EGFR* mutations. In addition, we found that high VEGFC expression was significantly correlated with poor survival (OS [*P* = 0.03] ([Fig 3a] and PFS [*P* = 0.002] ([Fig 2b]) in patients with stage I/II, similarly, in stage III with poor OS (*P* = 0.02) (Fig 3c) and PFS (*P* = 0.02) (Fig 3d). Finally, high VEGFC expression was significantly correlated with an adverse OS (*P* = 0.003) (Fig 3e) and PFS (*P* = 0.01) (Fig 3f) in acinar adenocarcinoma, and a poor PFS (*P* = 0.01) (Fig 3h) in nonacinar adenocarcinoma.

**Figure 4:**
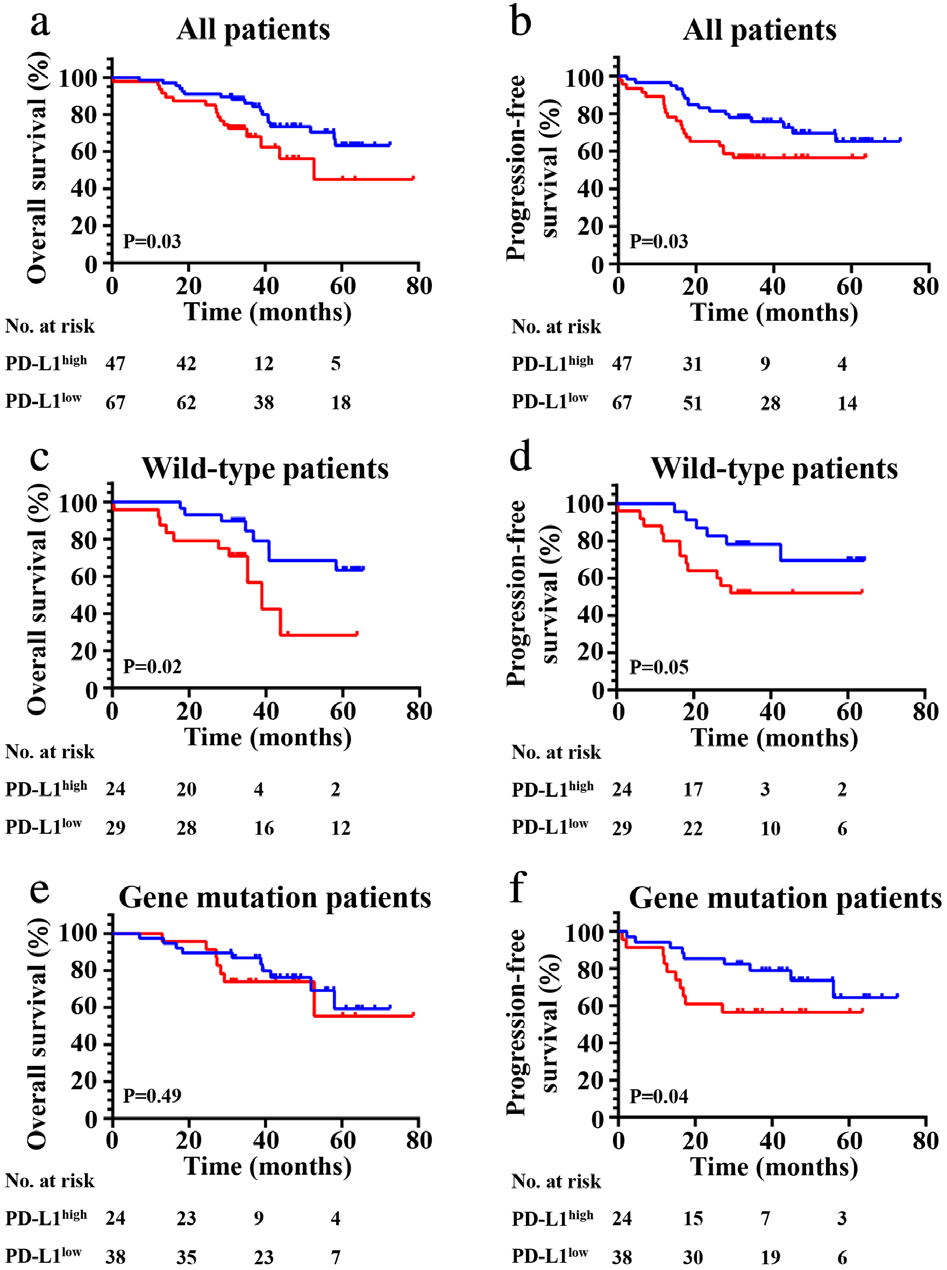
Kaplan‐Meier curves show (**a**, **c** and **e**) OS and (**b**, **d** and **f**) PFS of different groups (all patients, wild‐type patients or gene mutation patients, respectively) with high and low expression of PD‐L1. (**a**) (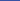) PD‐L1^low^ (*N* = 67, 18 events), and (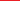) PD‐L1^high^ (*N* = 47, 17 events); (**b**) (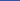) PD‐L1^low^ (*N* = 67, 17 events), and (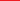) PD‐L1^high^ (*N* = 47, 20 events); (**c**) (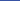) PD‐L1^low^ (*N* = 29, eight events), and (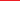) PD‐L1^high^ (*N* = 24, 10 events); (**d**) (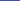) PD‐L1^low^ (*N* = 29, six events), and (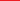) PD‐L1^high^ (*N* = 24, 12 events); (**e**) (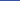) PD‐L1^low^ (*N* = 38, 10 events), and (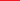) PD‐L1^high^ (*N* = 24, seven events); (**f**) (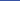) PD‐L1^low^ (*N* = 38, eight events), and (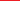) PD‐L1^high^ (*N* = 24, 11 events).

**Figure 5:**
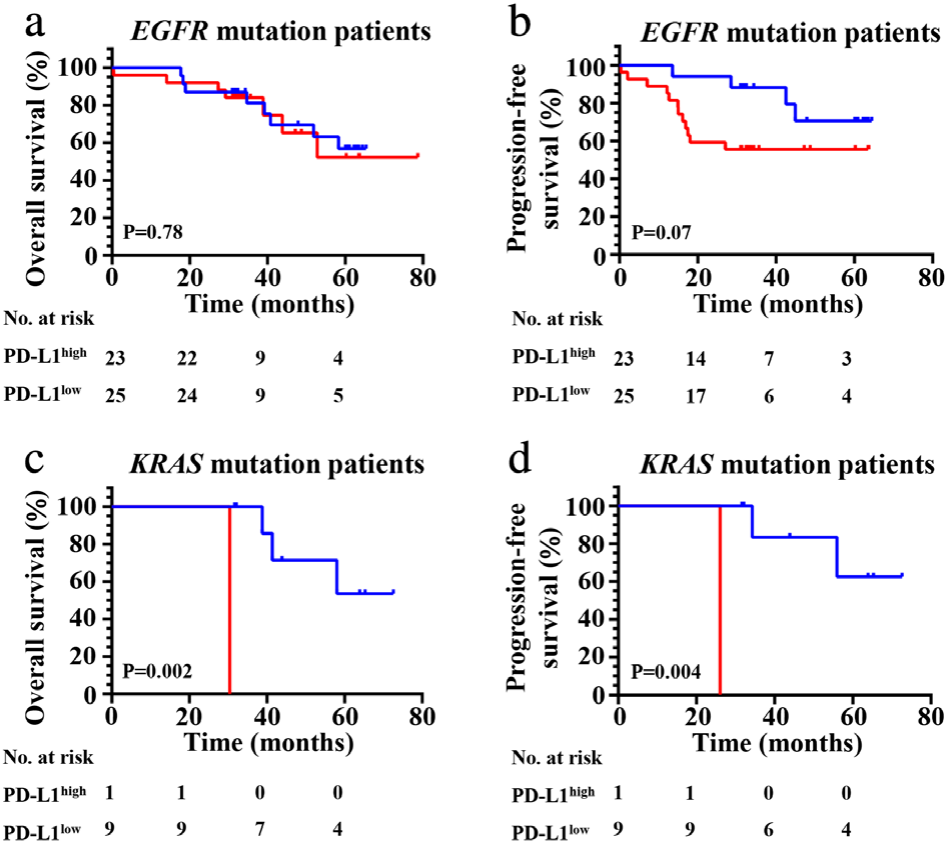
Kaplan‐Meier curves show (**a** and **c**) OS and (**b** and **d**) PFS of different subgroups (*EGFR* mutation patients or *KRAS* mutation patients, respectively) with high and low expression of PD‐L1. (**a**) (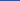) PD‐L1^low^ (*N* = 23, eight events), and (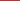) PD‐L1^high^ (*N* = 25, seven events); (**b**) (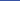) PD‐L1^low^ (*N* = 23, four events), and (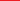) PD‐L1^high^ (*N* = 25, 12 events); (**c**) (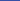) PD‐L1^low^ (*N* = 9, three events), and (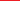) PD‐L1^high^ (*N* = 1, one event); (**d**) (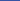) PD‐L1^low^ (*N* = 9, two events), and (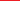) PD‐L1^high^ (*N* = 1, one event).

**Figure 6:**
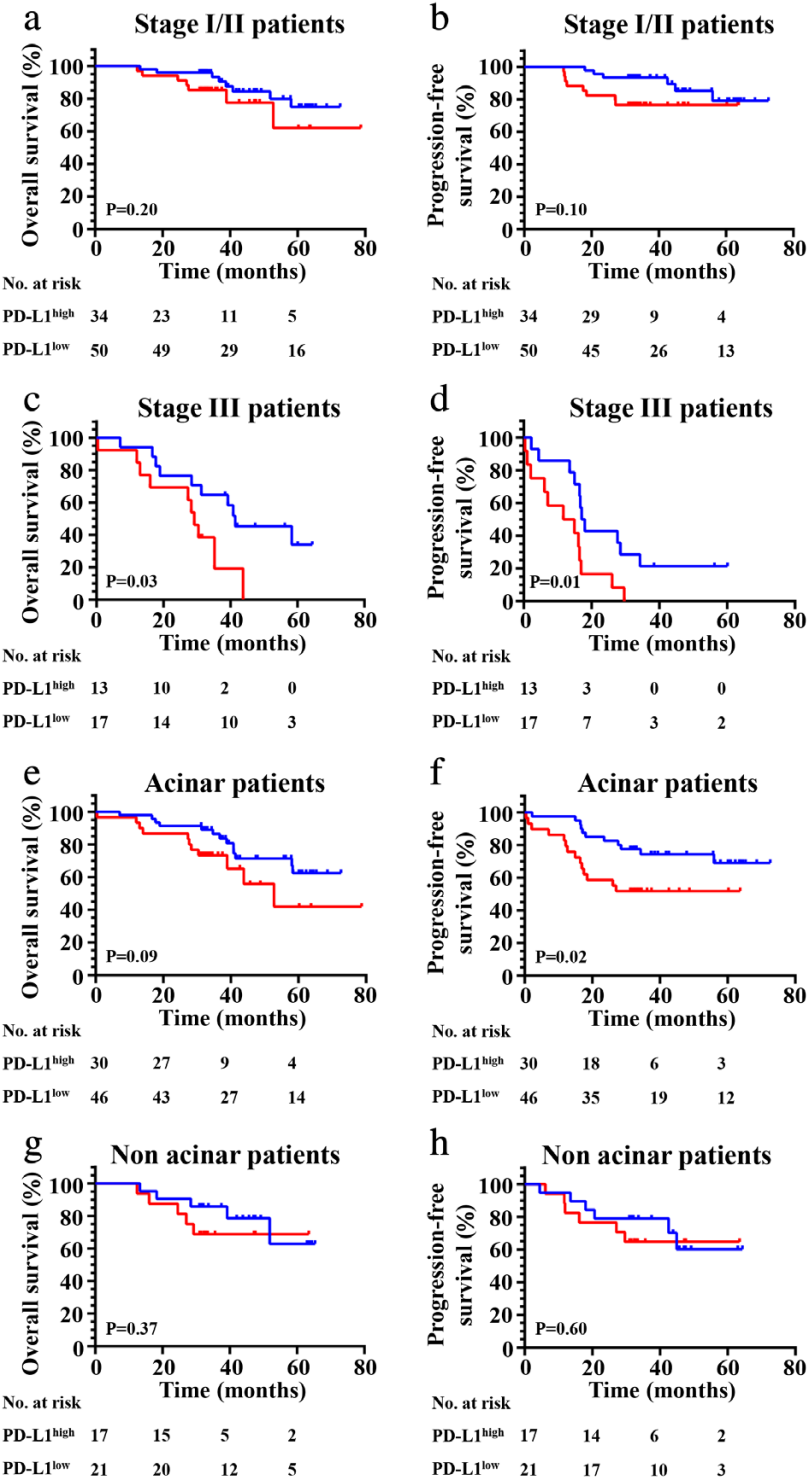
Kaplan‐Meier curves show (**a**, **c**, **e** and **g**) OS and (**b**, **d**, **f** and **h**) PFS of different subgroups (clinical stage I/II, III acinar or nonacinar adenocarcinoma, respectively) with high and low expression of PD‐L1. (**a**) (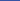) PD‐L1^low^ (*N* = 50, eight events), and (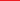) PD‐L1^high^ (*N* = 34, seven events); (**b**) (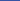) PD‐L1^low^ (*N* = 50, six events), and (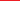) PD‐L1^high^ (*N* = 34, eight events); (**c**) (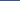) PD‐L1^low^ (*N* = 17, 10 events), and (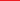) PD‐L1^high^ (*N* = 13, 10 events); (**d**) (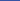) PD‐L1^low^ (*N* = 17, 11 events), and (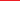) PD‐L1^high^ (*N* = 13, 12 events); (**e**) (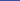) PD‐L1^low^ (*N* = 46, 13 events), and (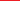) PD‐L1^high^ (*N* = 30, 11 events); (**f**) (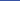) PD‐L1^low^ (*N* = 46, 11 events), and (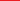) PD‐L1^high^ (*N* = 30, 14 events); (**g**) (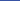) PD‐L1^low^ (*N* = 21, five events), and (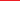) PD‐L1^high^ (*N* = 17, five events); (**h**) (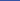) PD‐L1^low^ (*N* = 21, six events), and (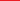) PD‐L1^high^ (*N* = 17, six events).

Moreover, we found high PD‐L1 expression was significantly correlated with shorter OS (*P* = 0.03) (Fig 4a) and PFS (*P* = 0.03) (Fig 4b) when considering all patients in our cohort. Prognostic analysis in relation to PD‐L1 expression was then performed in the different subgroups such as VEGFC. First, we found high PD‐L1 expression exhibited a poor OS (*P* = 0.02) (Fig 4a,c) and PFS (*P* = 0.05) (Fig 4d) when compared with patients in the PD‐L1 low expression group in wild‐type patients. Second, no significant correlation between high PD‐L1 expression and OS (Fig 4e) (*P* = 0.0.49) was found, but an obvious relationship was found between high PD‐L1 expression and PFS (*P* = 0.04) (Fig 4f) in patients with any gene mutation (*EGFR*, *KRAS* or *ALK*). Third, we found that high PD‐L1 expression had no important influence on OS (*P* = 0.78) (Fig 5a) and PFS (*P* = 0.07) (Fig 5b) in patients with *EGFR* mutations. However, we found high PD‐L1 expression had a significant impact on OS (*P* = 0.002) (Fig 5c) and PFS (*P* = 0.004) (Fig 5d) in patients with *KRAS* mutations. Finally, we found high PD‐L1 expression was not significantly correlated with adverse survival in patients with clinical stage I/II (Fig 6a,b), and in contrast was significantly correlated with poor OS (*P* = 0.03) (Fig 6c) and PFS (*P* = 0.01) (Fig 6d) in stage III. The same results were found in patients with acinar adenocarcinoma wherein high PD‐L1 expression was significantly correlated with an adverse PFS (*P* = 0.02) (Fig 6f), but not to poor OS (*P* = 0.37) (Fig 6g) or PFS (*P* = 0.60) (Fig 6(h)) in patients with nonacinar adenocarcinoma.

**Figure 7:**
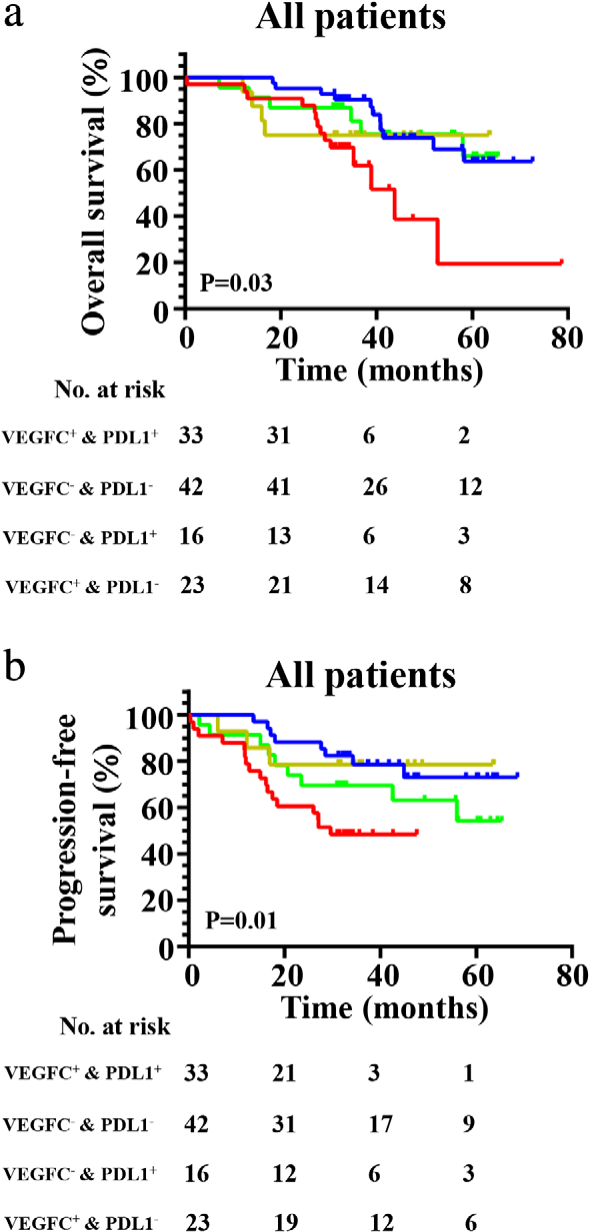
Kaplan‐Meier curves show (**a**) OS (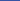) VEGFC^+^ & PDL1^+^ (*N* = 33, 14 events), (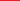) VEGFC^–^ & PDL1^–^ (*N* = 42, 11 events), (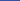) VEGFC^–^ & PDL1^+^ (*N* = 16, four events), and (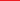) VEGFC^+^ & PDL1^–^ (*N* = 23, six events) and (**b**) PFS in patients with VEGFC^−^& PD‐L1^−^, VEGFC^+^& PD‐L1^−^, VEGFC^−^& PD‐L1^+^ and VEGFC^+^& PD‐L1^+^ expression (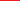) VEGFC^+^ & PDL1^+^ (*N* = 33, 17 events), (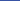) VEGFC^–^ & PDL1^–^ (*N* = 42, eight events), (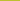) VEGFC^–^ & PDL1^+^ (*N* = 16, three events), and (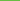) VEGFC^+^ & PDL1^–^ (*N* = 23, nine events).

Finally, we conducted combinatory analysis of VEGFC and PD‐L1 and found that the VEGFC^+^& PD‐L1^+^ group had worse OS (*P* = 0.03) (Fig 7a) and PFS (*P* = 0.01) (Fig 5b) when compared to the other three groups (VEGFC^−^& PD‐L1^−^, VEGFC^+^& PD‐L1^−^ or VEGFC^−^& PD‐L1^+^). Additionally, there were no clinical features associated with VEGFC/PD‐L1 co‐expression (Table S1 and S2).

In our univariate analysis on all lung adenocarcinoma patients, six clinicopathological characteristics were considered to be adverse prognostic factors for PFS: advanced T factor and N factor (>T2; >N0; all *P* < 0.001); clinical stage III (HR = 10.661 [95% CI 5.394–21.071], *P* < 0.0001); high VEGFC expression (HR = 0.370 [95% CI 0.182–0.375], *P* = 0.004); high PD‐L1 expression (HR = 1.979 [95% CI 1.030–3.800], *P* = 0.037), and VEGFC/PD‐L1 co‐expression (HR = 2.749 [95% CI 1.410–5.361], *P* = 0.002). These factors were also determined as poor prognostic factors for OS. In our multivariate analysis, high expression of VEGFC was an unfavorable prognostic factor for PFS (HR = 2.816 [95% CI 1.058–7.495], *P* = 0.038), and stage III was an adverse factor for both OS (HR = 3.516 [95% CI 1.278–9.679], *P* = 0.015) and PFS (HR = 8.884 [95% CI 3.287–24.015], *P* < 0.0001) (Table 3).

**Table 3:** Univariate and multivariate cox analysis of factors for progression‐free survival and overall survival in patients with lung adenocarcinoma (SEM)

Univariate cox analysis						
Variable	Overall survival			Progression‐free survival		
	HR	95% CI	*P*‐value	HR	95% CI	*P*‐value
Gender (female vs. male)	1.407	0.723–2.740	0.313	1.157	0.607–2.206	0.657
Age (<60 vs. ≥60)	1.596	0.819–3.108	0.166	1.227	0.642–2.344	0.535
Smoking history (no vs. yes)	1.121	0.576–2.183	0.737	0.958	0.496–1.850	0.899
T factor (T ≤ 2 vs. T > 2)	5.196	2.419–11.161	**<0.0001** ****	5.322	2.466–11.487	**<0.0001** ****
**N factor (N0 vs. > N0)	3.052	1.560–5.969	**0.001** ***	5.373	2.774–10.405	**<0.0001** ***
Gene mutations (wild‐type vs. *EGFR*)	0.958	0.472–1.948	0.907	1.040	0.530–2.040	0.910
Histological subtypes (acinar vs. nonacinar)	1.037	0.507–2.123	0.920	0.914	0.458–1.821	0.797
Stage (I/II vs. III)	4.935	2.522–9.656	**<0.0001** ****	10.661	5.394–21.071	**<0.0001** ****
VEGFC expression (≤100 vs. >100)	0497	0.251–0.983	**0.041** *	0.370	0.182–0.753	**0.004** ***
PD‐L1 expression (≤100 vs. >100)	2.038	1.035–4.014	**0.036** *	1.979	1.030–3.800	**0.037** *
Co‐expression	2.761	1.360–5.605	**0.004** ***	2.749	1.410–5.361	**0.002** ***
Multivariate cox analysis						
T factor	2.448	0.983–6.098	0.054	1.207	0.497–2.935	0.678
N factor	1.339	0.536–3.344	0.532	1.507	0.623–3.649	0.363
Stage	3.516	1.278–9.679	**0.015** *	8.884	3.287–24.015	**<0.0001** ****
VEGFC expression	2.150	0.799–5.787	0.130	2.816	1.058–7.495	**0.038** *
PD‐L1 expression	2.260	0.666–7.673	0.191	2.893	0.932–8.984	0.066
Co‐expression	0.656	0.135–3.176	0.600	0.501	0.124–2.019	0.331

* P=0.05

** P=0.01

*** P=0.001

**** P=0.0001.

## Discussion

Emerging evidence indicates that VEGF‐C can modulate the immune system to facilitate tumor cells to more easily escape immune surveillance.^5^, ^6^, ^7^, ^21^, ^22^ In addition, PD‐L1 can also promote tumor cells to escape from host immune attack.^23^, ^24^ To the best of our knowledge, the present research is the first study which exposes VEGFC and PD‐L1 expression in patients with lung adenocarcinoma and evaluates the relationship between their expression and prognosis of lung adenocarcinoma.

First, we found high VEGFC expression was significantly associated with poor survival in patients with lung adenocarcinoma, which is concordant with the results of other studies.^25^, ^26^ In our study, further analysis showed that in the gene mutations subgroup (patients with one of any three mutated genes) and stage I/II, stage III, acinar and nonacinar, there was a significant association between high expression of VEGFC and poor PFS.

Similar to VEGFC expression, we discovered PD‐L1 high expression was closely related to poor survival in all patients. When analyzed in the subgroups, there was a significant association between PD‐L1 expression and PFS in patients with any gene mutation cohort wherein mutant *EGFR* accounted for 48/62 (77.4%), mutant *KRAS* accounted for 10/62 (16.1%) and mutant *ALK* accounted for 4 /62 (6.5%). Further analysis showed that the *KRAS* mutation subgroup (16.1%) caused the statistical significance in the gene mutation population; however, high PD‐L1 expression had no significant correlation to poor PFS in patients in the *EGFR* mutation group. In addition, high PD‐L1 expression predicted a worse PFS in the *EGFR* wild‐type, stage III and acinar groups, but not in the stage I/II and nonacinar groups. A meta‐analysis of three randomized phase 2 or 3 studies (CheckMate057, POPLAR, and OAK) found that patients with *KRAS* mutations represented a survival benefit from immune checkpoint inhibitors (ICIs: anti‐PD‐1/PD‐L1 therapy) compared with other treatment.^27^ Another meta‐analysis including three clinical trials (CheckMate057, POPLAR, and KEYNOTE‐010) discovered that *EGFR*‐mutated patients did not gain a survival benefit from ICIs compared with other treatment.^28^ The *KRAS* mutation group might have caused the statistical significance of PFS in the mutant gene population. Additionally, high PD‐L1 expression was associated with poor OS and PFS in lung cancer patients with acinar adenocarcinoma and clinically diagnosed stage III lung cancer patients, and this has also been confirmed in other studies.^29^, ^30^

In addition, we confirmed for the first time that VEGFC^+^& PD‐L1^+^ patients (28.9%, 33/114) had a worse PFS and OS among all four types (VEGFC^−^& PD‐L1^−^, VEGFC^−^& PD‐L1^+^, VEGFC^+^& PD‐L1^−^ and VEGFC^+^& PD‐L1^+^). In addition, VEGFC expression was positively related to PD‐L1 expression. The lymphatic vasculature is critical to immunity with one of its major roles being the trafficking of immune cells.^31^ High VEGF‐C expression in experimental mouse models has previously been found to promote lymphatic vessel enlargement with lymphatic endothelial cell proliferation.^32^, ^33^ Recently, several studies have found that dilated lymphatic vessels exhibit impaired transport capacity,^33^, ^34^ making immune cells difficult to transport into the tumor. Simultaneously, high expression of PD‐L1 could enhance tumor immune evasion and has been associated with poor survival in various malignancies including lung cancer.^16^ Therefore, even if a few immune cells infiltrate into tumor tissue through abnormal lymphatic vasculature, tumor cells with high PD‐L1 expression would inhibit activation of immune cells. Conclusively, co‐expression of PD‐L1 and VEGFC will be a predictor for high recurrent risk and poor prognosis. For those patients, the combination of anti‐VEGFC and anti‐PDL1 could be a synergistic effective treatment strategy. It would also provide a theoretical possibility for screening optimal population with a combination of anti‐VEGFC and anti‐PD‐L1 therapy. We also analyzed the relationship between co‐expression of PD‐L1 and VEGFC and clinical features, but failed to find a significant correlation, possibly due to the small pool of patient samples.

There are several limitations to this study. First, it was retrospective and conducted in our hospital with a small pool of patient samples. Second, it should be emphasized that this was an initial and immature study. Third, the underlying mechanisms need to be highlighted in future investigations.

In conclusion, both high VEGFC and PD‐L1 expression indicate a poor prognosis in lung adenocarcinoma patients, and VEGFC is positively correlated with PD‐L1. Furthermore, co‐expression of VEGFC and PD‐L1 led to a significantly worse prognosis among all four types (VEGFC^−^& PD‐L1^−^, VEGFC^−^& PD‐L1^+^, VEGFC^+^& PD‐L1^−^ and VEGFC^+^& PD‐L1^+^). In the future, VEGFC and PD‐L1 co‐expression may therefore be used as a prognostic indicator for the clinical outcome. In addition, our study also provides the theoretical possibility to screen the optimal population with a combination of anti‐VEGFC and anti‐PD‐L1 therapy in lung adenocarcinoma.

## Disclosure

The authors declare that they have no known competing financial interests or personal relationships which have, or could be perceived to have, influenced the work reported in this article.

## Supporting information


**Figure S1** Scatter diagram showing the correlation of VEGFC expression and PD‐L1 expression based on the results of H‐score.
**Table S1** Correlation of expression of VEGFC and/or PD‐L1 and the clinical characteristics in 114 patients with lung adenocarcinoma (SEM).
**Table S2** Correlation of co‐expression of VEGFC and PD‐L1 and the clinical characteristics in 114 patients with lung adenocarcinoma (SEM).
