# Supplementary material for: Clinical importance of VEGFC and PD‐L1 co‐expression in lung adenocarcinoma patients
Source: Thorac Cancer. 2020 Mar 10;11(5):1139–48. doi: 10.1111/1759-7714.13354 (PMC7180596; doi:10.1111/1759-7714.13354)
Supplement: Supplementary file 1 — Figure S1 Scatter diagram showing the correlation of VEGFC expression and PD‐L1 expression based on the results of H‐score. Table S1 Correlation of expression of VEGFC and/or PD‐L1 and the clinical characteristics in 114 patients with lung adenocarcinoma (SEM). Table S2 Correlation of co‐expression of VEGFC and PD‐L1 and the clinical characteristics in 114 patients with lung adenocarcinoma (SEM). [file TCA-11-1139-s001.docx]

Supplementary Material

## Supplementary Figures


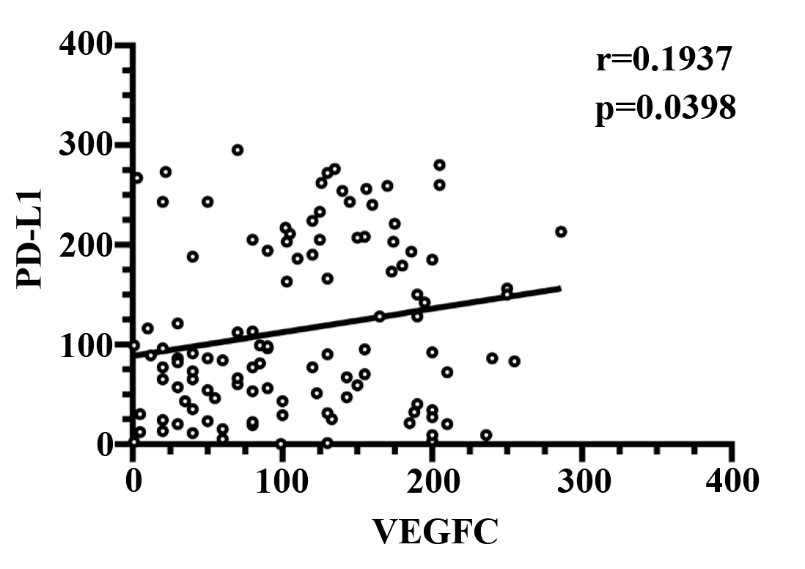


**Supplement figure 1.** Scatter diagram showing the correlation of VEGFC expression and PD-L1 expression based on the results of H-score.

**Supplement table 1. Correlation of expression of PD-L1 and/or VEGFC and the clinical characteristics in 114 patients with LADC.**

|  | **PD-L1^-^/VEGFC^-^, n (%)** | **PD-L1^-^/VEGFC^+^, n (%)** | **PD-L1^+^/VEGFC^-^, n (%)** | **PD-L1^+^/VEGFC^+^, n (%)** | **P -value** |
| --- | --- | --- | --- | --- | --- |
| **T factor** |  |  |  |  | 0.257 |
| T2≤ | 39(92.9) | 23(95.8) | 14(93.3) | 27(81.8) |  |
| ＞T2 | 3(7.1) | 1(4.2) | 1(6.7) | 6(18.2) |  |
| **N factor** |  |  |  |  | 0.486 |
| N0 | 28(66.7) | 19(79.2) | 11(73.3) | 20(60.6) |  |
| >N0 | 14(33.3) | 5(20.8) | 4(26.7) | 13(39.4) |  |
| **Clinical stage** |  |  |  |  | 0.678 |
| Ⅰ/Ⅱ | 31(73.8) | 19(79.2) | 12(80.0) | 22(66.7) |  |
| Ⅲ | 11(26.2) | 5(20.8) | 3(20.0) | 11(33.3) |  |
| **Gender** |  |  |  |  | 0.795 |
| Male | 18(42.9) | 13(54.2) | 6(40.0) | 15(45.5) |  |
| Female | 24(57.1) | 11(45.8) | 9(60.0) | 18(54.5) |  |
| **Age** |  |  |  |  | 0.144 |
| ＜60 | 25(59.5) | 17(70.8) | 10(66.7) | 14(42.4) |  |
| ≥60 | 17(40.5) | 7(29.2) | 5(33.3) | 19(57.6) |  |
| **Smoking history** |  |  |  |  | 0.342 |
| Yes | 26(61.9) | 10(41.7) | 9(60.0) | 21(63.6) |  |
| No | 16(38.1) | 14(58.3) | 6(40.0) | 12(36.4) |  |
| **Acianr adenocarcinoma** |  |  |  |  | 0.808 |
| Yes | 30(71.4) | 16(66.7) | 10(66.7) | 20(60.6) |  |
| No | 12(28.6) | 8(33.3) | 5(33.3) | 13(39.4) |  |
| **EGFR mutations** |  |  |  |  | 0.255 |
| Yes | 14(33.3) | 9(62.5) | 9(60.0) | 16(51.5) |  |
| No | 28(66.7) | 15(37.5) | 6(40.0) | 17(48.5) |  |

**Supplement table 2. Correlation of co-expression of PD-L1 and VEGFC and the clinical characteristics in 114 patients with LADC**

|  | **Without PD-L1^+^/VEGFC^+^, n (%)** | | **With**  **PD-L1^+^/VEGFC^+^, n (%)** | **P-value** | **PD-L1^-^/VEGFC^-^, n (%)** | | **PD-L1^+^/VEGFC^+^, n (%)** | **P-value** |
| --- | --- | --- | --- | --- | --- | --- | --- | --- |
| **T factor** | |  |  | 0.051 |  |  | | 0.144 |
| T2≤ | | 76 93.8) | 27 (81.8) |  | 39 (92.9) | 27 (81.8) | |  |
| ＞T2 | | 5 (6.2) | 6 (18.2) |  | 3 (7.1) | 6 (18.2) | |  |
| **N factor** | |  |  | 0.252 |  |  | | 0.587 |
| N0 | | 58 (71.6) | 20 (60.6) |  | 28 (66.7) | 20 (60.6) | |  |
| >N0 | | 23 (28.4) | 13 39.4) |  | 14 (33.3) | 13 (39.4) | |  |
| **Clinical stage** | |  |  | 0.277 |  |  | | 0.500 |
| Ⅰ/Ⅱ | | 62 (76.5) | 22 (66.7) |  | 31 (73.8) | 22 (66.7) | |  |
| Ⅲ | | 19 (23.5) | 11 (33.3) |  | 11 (26.2) | 11 (33.3) | |  |
| **Gender** | |  |  | 0.983 |  |  | | 0.822 |
| Male | | 37 (45.7) | 15 (45.5) |  | 18 (42.9) | 15 (45.5) | |  |
| Female | | 44 (54.3) | 18 (54.5) |  | 24 (57.1) | 18 (54.5) | |  |
| **Age** | |  |  | 0.065 |  |  | | 0.141 |
| ≤60 | | 52 (64.2) | 14 (42.4) |  | 25 (59.5) | 14 (42.4) | |  |
| ＞60 | | 29 (35.8) | 19 (57.6) |  | 17 (40.5) | 19 (57.6) | |  |
| **Smoking history** | |  |  | 0.428 |  |  | | 0.878 |
| Yes | | 45 (55.6) | 21 (63.6) |  | 26 (61.9) | 21 (63.6) | |  |
| No | | 36 (44.4) | 12 (36.4) |  | 16 (38.1) | 12 (36.4) | |  |
| **EGFR mutations** | |  |  | 0.379 |  |  | | 0.184 |
| Yes | | 32 (39.5) | 16 (48.5) |  | 14 (33.3) | 16 (48.5) | |  |
| No | | 49 (60.5) | 17 (51.5) |  | 28 (66.7) | 17 (51.5) | |  |
| **Acinar adenocarcinoma** | |  |  | 0.381 |  |  | | 0.324 |
| Yes | | 56 (69.1) | 20 (60.6) |  | 30 (71.4) | 20 (60.6) | |  |
| No | | 25 (30.9) | 13 (39.4) |  | 12 (28.6) | 13 (39.4) | |  |
